# Adiponectin in Myopathies

**DOI:** 10.3390/ijms20071544

**Published:** 2019-03-27

**Authors:** Tania Gamberi, Francesca Magherini, Tania Fiaschi

**Affiliations:** Dipartimento di scienze Biomediche, Sperimentali e Cliniche “M. Serio”, Università degli studi di Firenze, Viale Morgagni 50, 50134 Firenze, Italy; tania.gamberi@unifi.it (T.G.); francesca.magherini@unifi.it (F.M.)

**Keywords:** adiponectin, muscle, myopathies

## Abstract

In skeletal muscle, adiponectin has varied and pleiotropic functions, ranging from metabolic, anti-inflammatory, insulin-sensitizing to regenerative roles. Despite the important functions exerted by adiponectin, the study of the hormone in myopathies is still marginal. Myopathies include inherited and non-inherited/acquired neuromuscular pathologies characterized by muscular degeneration and weakness. This review reports current knowledge about adiponectin in myopathies, regarding in particular the role of adiponectin in some hereditary myopathies (as Duchenne muscular dystrophy) and non-inherited/acquired myopathies (such as idiopathic inflammatory myopathies and fibromyalgia). These studies show that some myopathies are characterized by decreased concentration of plasma adiponectin and that hormone replenishment induces beneficial effects in the diseased muscles. Overall, these findings suggest that adiponectin could constitute a future new therapeutic approach for the improvement of the abnormalities caused by myopathies.

## 1. Introduction

### 1.1. Adiponectin in Skeletal Muscle

In skeletal muscle, adiponectin exerts several and pleiotropic biological effects, including the involvement in cellular metabolism that has been immediately evident since its discovery [1]. Adiponectin is mainly produced by adipose tissue as “full-length” (fAd) form, which can associate to form complexes circulating in the plasma. Circulating adiponectin oligomers comprise High Molecular Weight (HMW), Medium Molecular Weight (MMW), and Low Molecular Weight (LMW) forms [2]. fAd can be enzymatically cleaved to the smaller “globular form” (gAd) by the elastase produced by monocytes [3] or macrophages [4]. Skeletal muscle expresses two unusual, seven-transmembrane-spanning, and G-protein-independent adiponectin receptors, AdipoR1 and AdipoR2 [1]. The metabolic effects of adiponectin occur through the activation of intracellular signalling pathways initiated by the binding with the adiponectin receptors of the adaptor protein containing pleckstrin homology domain, phosphotyrosine binding domain, and leucine zipper domain (APPL1) [5]. APPL1 plays a crucial role in adiponectin-mediated effects, as the recruitment of glucose transporter GLUT4 to the plasma membrane [6] and the activation of AMP kinase (AMPK) [7]. Full AMPK activation occurs through both phosphorylation by liver kinase B1 (LKB1) and AMP binding [8]. In skeletal muscle, AMPK induces the inhibitory phosphorylation of Acetyl-CoA carboxylase (ACC), leading to decreased formation of malonyl CoA [9], activation of oxidation, and inhibition of fatty acid synthesis [9]. Moreover, adiponectin-dependent fatty acid oxidation in skeletal muscle occurs also through the activation of p38 MAPK and PPARα signalling pathways [10]. In addition, adiponectin regulates mitochondrial biogenesis through the binding with AdipoR1. This event leads to the activation and increased expression of peroxisome proliferator-activated receptor gamma coactivator 1-alpha (PGC-1α), which promotes mitochondrial biogenesis, the increase of oxidative metabolism, and formation of type I myofibers [11].

In skeletal muscle, adiponectin exerts an insulin-sensitizing role in which the decreased intracellular lipid content induced by the hormone is deeply involved [12]. Among the several types of lipids, elevated intracellular levels of ceramide have been reported to have cellular deleterious effects and greatly contribute to insulin resistance [13,14,15]. Adiponectin decreases intracellular ceramide content through the activation of ceramidase activity associated to AdipoR1/AdipoR2. Ceramide is then converted in sphingosine, which, in turn, became phosphorylated to sphingosine 1-phosphate (S1P) due to sphingosine kinase. Sphingosine and S1P are involved in PPARα and AMPK activation, respectively, thus leading to lipid oxidation, mitochondrial biogenesis, and glucose utilization [15,16,17]. This mechanism has been suggested to be involved in insulin sensitivity, since it decreases cellular availability of sphingolipid precursors and therefore enhances insulin signalling due to reduced ceramide content [17]. In addition, the sphingolipid-mediated pathway, involving probably ceramidase activity, has been reported to be involved in blocking apoptosis in cardiomyocytes [17].

### 1.2. Adiponectin Is a Myokine and a Myogenic Factor

Alongside adipose tissue, which secretes in the blood stream endocrine adiponectin through a largely elucidated molecular mechanism [18,19,20], a local secretion of the hormone has been reported by several tissues [21], including skeletal muscle. Several papers described skeletal muscle as secretory organ [22,23] able to locally secrete adiponectin [4,24,25,26,27]. In skeletal muscle, myotubes enhance the secretion of fAd in inflamed or pro-oxidant microenvironment [26,27] that is generated by a trauma. This condition could lead to the recruitment of macrophage which participate to the cleavage of fAd into gAd [4].

Besides the metabolic and insulin-sensitizing role, adiponectin acts as a myogenic factor through the participation in muscle differentiation and tissue regeneration, and influencing the behavior of muscle cells. Adiponectin acts on satellite cells, a population of stem cells involved in muscle regeneration in adult skeletal muscles, which undergo activation following trauma [28]. Adiponectin promotes satellite cell activation through the activation of the p38 MAPK signalling cascade [4]. In addition, adiponectin induces the expression of the transcription factors Snail and Twist, responsible for the activation of a motile program, thus permitting satellite cells to reach the site of damage. Cell motility induced by adiponectin involves the enhancement of metalloproteinase-2 secretion, thus facilitating the arrival of satellite cells to damaged site by degrading extracellular matrix [4]. In vitro, adiponectin acts as a myogenic factor both in myoblasts and in mesoangioblasts. In myoblasts, adiponectin induces the exit of cells from cell cycle and promotes myotubes formation [27]. We reported that adiponectin in myoblasts activates autophagy and that this autophagic process is strictly associated with the myogenic role of the hormone. Indeed, the inhibition of autophagy leads to the impairment of myotube formation due to adiponectin. These in vitro results were confirmed on adiponectin-KO mice, that showed decreased autophagy markers in skeletal muscle and a myopathic phenotype, thus demonstrating a close correlation between activation of autophagy and the differentiating role of adiponectin in skeletal muscle [29]. In addition to the role on resident muscle cells, as satellite cells and myoblasts, adiponectin also acts on the non-resident muscle precursors, mesoangioblasts [30]. These are multipotent cells capable of differentiation towards myogenic lineage and that gave promising results in gene therapy for the treatment of Duchenne muscular dystrophy [31,32]. Where mesoangioblasts are concerned, adiponectin affects in vitro several cellular features as the increased mesoangioblast migration towards myotubes, the enhancement of cell survival upon growth factor withdrawal or extracellular matrix detachment and promotes myogenesis [33]. The ex vivo treatment of mesoangioblasts with adiponectin and the following injection of treated cells into dystrophic muscles of sarcoglycan-null mice ameliorates in vivo mesoangioblast survival and improves their engraftment in the diseased muscles [33].

Decreased plasma adiponectin levels have been associated to different pathologies, including obesity and type 2 diabetes [34,35,36]. Obesity induced endoplasmic reticulum stress and impaired unfolded protein response in adipocytes, and both mechanisms seem to be responsible for the diminished adiponectin secretion in obese mice [37,38]. Hypoadiponectinemia alters several functions of skeletal muscle, such as glucose and lipid metabolism and muscle regeneration [39,40]. Indeed, obese mice display diminished regenerative capacity of skeletal muscle following injury, probably due to a reduced macrophage recruitment and angiogenesis [39], increased lipid accumulation and pro-inflammatory cytokines, and impaired satellite cell activity [40].

## 2. Adiponectin in Myopathies

Although the key role of adiponectin in healthy skeletal muscle has been well established, the study of the hormone in myopathies is just getting started. Myopathies refer to neuromuscular disorders of skeletal muscles characterized by muscular degeneration and weakness. Myopathies may be classified into two main categories: inherited and non-inherited/acquired myopathies. Inherited myopathies include muscular dystrophies, congenital myopathies, mitochondrial myopathies, and metabolic myopathies. Non inherited/acquired myopathies comprise inflammatory myopathies, toxic myopathies, and myopathies associated with systemic conditions [41].

**Figure 1:**
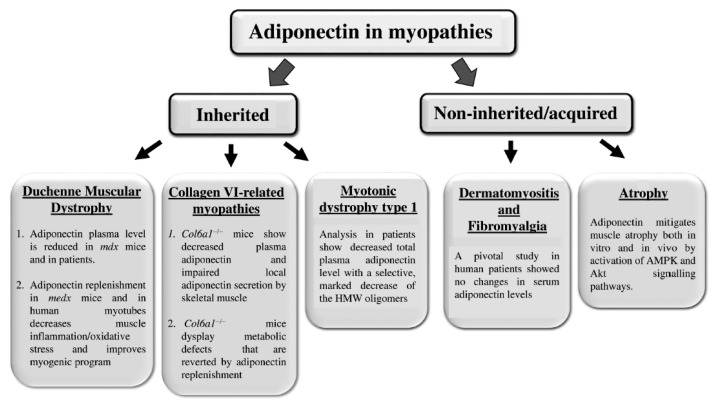
The state of the art about adiponectin in myopathies. Each panel reports the results obtained in the different myopathies (inherited and not inherited/acquired) about adiponectin. More details are explained in the text.

Figure 1 summarizes the main results obtained on adiponectin in both inherited and non-inherited/acquired myopathies.

### 2.1. Adiponectin in Inherited Myopathies

Muscular dystrophies are inherited disorders, triggered by a genetic mutation that typically affects striated muscle tissue. Duchenne muscular dystrophy (DMD) is an X-linked recessive defect caused by dystrophin gene mutation. Dystrophin is a key scaffolding protein of the dystroglycan complex [42], which connects the myofibers to cytoskeleton and the extracellular matrix. Dystroglycan complex injuries lead to sarcolemma instability and vulnerability to mechanical stress, [43] thus permitting the infiltration of immune cells and generating inflammation, necrosis, and severe muscle degeneration. The chronic inflammation/oxidative stress plays a crucial role in DMD pathogenesis [44].

So far, most of the studies on adiponectin and dystrophies were mainly performed on mouse models. *mdx* mice, a widely used mouse model of DMD, show decreased plasma adiponectin level due to a reduced secretion of adiponectin by adipose tissue [45], probably as the result of the systemic inflamed and stressed environment present in *mdx* mice. Indeed, adiponectin secretion by adipose tissue is strictly dependent by stressed conditions. Decreased adiponectin level is associated with obesity [35], diabetes [34,36], and coronary artery disease [46], and is closely related with oxidative stress [38,47]. Impaired mitochondrial function in adipocytes induced endoplasmic reticulum stress, which leads to the activation of signalling pathways, involving c-Janus Kinase (JNK) and Cyclic AMP-dependent transcription factor (ATF)-3A, culminating in decreased adiponectin synthesis [20]. Replenishment of adiponectin, obtained by crossing *mdx* mice with transgenic mice moderately overexpressing adiponectin, counteracts muscle inflammation by reducing the expression of inflammation markers as Tumour Necrosis Factor (TNF) α and Interleukin (IL)-1β and upregulating the anti-inflammatory cytokine IL-10. Besides its anti-inflammatory properties, adiponectin also improves myogenic program as well as muscle function. Indeed, *mdx*–adiponectin mice displayed partial or complete restoration of the regulators of the early phase of differentiation MyoD, Myf5, as well as Mrf4 [45]. The importance of adiponectin in the physiology of the dystrophic muscle has been confirmed using adiponectin KO–*mdx* mice that displayed a worsened dystrophic phenotype. Conversely, reinsertion of adiponectin gene in the skeletal muscle of *mdx*–adiponectin KO mice lead to decreased expression levels of several oxidative stress/inflammatory markers as well as the activity of NF-κB, and the concomitant increased expression levels of the myogenic markers [48].

Studies performed on primary human cultures of myotubes from DMD patients confirmed the results obtained in *mdx* mice. In line with the studies on animal models, human dystrophic myotubes show a local decrease of adiponectin secretion [49]. Moreover, primary cultures of human myotubes isolated from DMD patients exposed to chronic inflammation, confirming the anti-inflammatory effects of adiponectin in skeletal muscle. This protective effect occurs through AdipoR1 binding and activation of AMPK-SIRT1-PGC-1α signalling pathway, thereby leading to NF-κB downregulation [45,49]. Analysis of the myokine secretion profile of DMD human myotubes treated with adiponectin following an inflammatory stimulus, pointed out the downregulation of several pro-inflammatory molecules (as TNFα, IL-17A, and CCL28) and the upregulation of anti-inflammatory IL6. Accordingly, adiponectin regulates the expression level of the NLRP3 inflammasome, which has been reported to be involved in the worsening of DMD [50]. DMD human myotubes expressed threefold increase of NLRP3 level in comparison to healthy myotubes, and their treatment with adiponectin or with miR-711—considered a strong candidate for the adiponectin anti-inflammatory action [51]—attenuates NLRP3 inflammasome expression level [50]. Concerning circulating adiponectin in DMD patients, a single study reports the increase with age of plasma adiponectin [52].

Inherited myopathies comprise the Collagen VI-related myopathies (COL6-RM). Collagen VI is one of the most abundant extracellular matrix proteins in adipose tissue [53,54,55] and its expression is positively regulated by glucose levels and negatively by PPAR-γ agonists and leptin [56,57]. COL6-RM refer to congenital muscular dystrophy caused by mutation in one of the human collagen VI genes (COL6A1, COL6A2, and COL6A3) and are characterized by a varied degree of muscle weakness and joint contractures. They include early severe forms (as Ullrich Congenital Muscular Dystrophy, UCMD), milder presentations (as Bethlem Myopathy, BM) and intermediate phenotypes [58].

Recently, we performed a study of adiponectin and collagen VI-related myopathies using collagen VI-null (*Col6a1*^−/−^) mice that display myopathic phenotype close to human patients, thus representing a good animal model for the study of these genetic disorders [59]. Our findings show that *Col6a1*^−/−^ mice have decreased plasma adiponectin and impaired local adiponectin secretion by skeletal muscle. We found *Col6a1*^−/−^ myoblasts display several metabolic abnormalities, including impaired glucose uptake, altered mitochondria membrane potential, associated with a decreased oxygen consumption. These metabolic defects are reverted by adiponectin replenishment that restores *Col6a1*^−/−^ metabolic properties close to that of the healthy myoblasts [60].

Where human samples are concerned, transcriptome analysis performed using skeletal muscle biopsies of UCMD patients pointed out an increase of the mRNA levels of the main adipokines (as leptin and adiponectin). However, this transcriptomic data has not been confirmed at the intracellular protein level due to the small number of patients available [61].

Myotonic dystrophy type 1 (DM1) is a rare genetic disorder characterized by muscle wasting and metabolic comorbidity and increased risk of developing insulin resistance (IR) and type 2 diabetes [62]. An analysis carried out in 21 DM1 patients revealed a decrease of total plasma adiponectin with a selective, marked decrease of the HMW oligomers. Although not yet proven, it has been hypothesized that the decreased adiponectin level might contribute to the worsening of IR and metabolic complications observed in DM1 patients [63].

### 2.2. Adiponectin in Non-Inherited/Acquired Myopathies

Non-inherited myopathies include idiopathic inflammatory myopathies (IIM), which refers to a heterogeneous group of autoimmune muscle disorders classified in four phenotypes: dermatomyositis (DM), polymyositis (PM), necrotizing autoimmune myositis, and inclusion-body myositis.

A pivotal study in DM and PM patients focused on the analysis of serum adipokine levels useful as markers of disease, showed no changes in adiponectin amount [64]. However, it has been reported a close correlation between serum adipokine levels and the onset of the metabolic syndrome in DM young female patients. This study reported that serum adiponectin levels are positively correlated with the onset of metabolic syndrome, which is highly prevalent in DM patients in relation to age and disease progression [65].

Adiponectin has also been studied in other types of non-hereditary myopathies. These include fibromyalgia, which is a disorder characterized by widespread musculoskeletal pain accompanied by fatigue, sleep, memory, and mood issues [66]. This study, planned to evaluate leptin and adiponectin levels in patients with fibromyalgia with or without overweight or obesity, showed no difference of adiponectin amount in comparison to healthy subjects [67].

Recently, an involvement of adiponectin in blocking muscle atrophy has been reported [68]. Muscle atrophy is caused by excessive protein breakdown associated to a decreased protein synthesis as a consequence of several pathologies like AIDS, cancer, renal and cardiac failure [69]. Adiponectin is able to mitigate muscle atrophy both in vitro and in vivo, and this beneficial effect occurs through the activation of AMPK and Akt signalling pathways [68].

## 3. Future Perspectives

Although just beginning, the study of adiponectin in myopathies highlights a possible role of the hormone in the ameliorations of the abnormalities observed in these diseases. These preliminary studies reinforce the idea that the study of adiponectin in myopathies must proceed. As some inherited myopathies, such as DMD, are associated with a decreased content of plasma adiponectin, the hormone could potentially be used as a marker for the onset of the pathology. So far, several studies explored adiponectin as a biomarker in different diseases including hepatitis C, various types of cancers, inflammation, renal disease, and atherosclerosis [70]. More importantly, adiponectin treatment induces in some myopathies the amelioration of the defects induced by the pathology. This finding opens the possibility about the use of adiponectin as a new tool for the improvement of abnormalities caused by muscular pathologies. Several efforts were performed towards the planning of new pharmacological therapies able to induce adiponectin beneficial effects in pathologic conditions. In 2013, Kadowaki’s group published a paper describing the discovery of an orally active synthetic small molecule (called AdipoRon) that binds to and activates both AdipoR1 and AdipoR2 receptors [71]. It has been reported that AdipoRon induces the same physiological effects of adiponectin in healthy tissues such as liver and skeletal muscle [71]. In addition, AdipoRon induces beneficial effects in some pathologic conditions as insulin resistance and type 2 diabetes in mice [71], cardiac disease induced by pressure overload [72], pancreatic cancer [73], liver injury by galactosamine [74], and diabetic nephropathy due to the decrease of ceramide content and lipotoxicity [75]. At the time, while gene therapy has not yet reached the desired results for the cure of congenital muscular myopathies, the treatment of myopathic patients with adiponectin or its agonists could be considered.
